# Supplementary material for: Influence of Lipomannan and Lipoarabinomannan Concentration on Mycobacterial Inner Membranes Characterized by All-atom Simulations
Source: bioRxiv. 2026 Apr 16:2026.04.15.718817. Preprint. [Version 1] doi: 10.64898/2026.04.15.718817 (PMC13104963; doi:10.64898/2026.04.15.718817)
Supplement: Supplement 1 [file media-1.pdf]

Supplementary Information

Influence of Lipomannan and Lipoarabinomannan Concentration on the Mycobacterial Inner Membrane Characterized by All-atom Simulations

Hwayoung Lee[1], Nathanlel Rygh[2], Matthieu Chavent[3]*, and Wonpil Im[1,2]*

[1]Department of Biological Sciences, Pennsylvania, 18015, USA

[2]Department of Bioengineering, Lehigh University, Pennsylvania, 18015, USA

[3]Centre de Intégrative (CBI), Laboratoire de Microbiologie et Génétique Moléculaires (LMGM), Université de Toulouse, CNRS, Toulouse 31400, France

*Corresponding authors: matthieu.chavent@utoulouse.fr and wonpil@lehigh.edu

Table S1. System composition and simulation details for the symmetric and asymmetric mycobacterial membrane models in this study.[1]

	Name	Abrev.	MPPE	MPPI	MPCL2	$AcPIM_6$	Ac_2PIM_6	$AcPIM_2$	Ac_2PIM_2	LM	LAM	CLA	POT	Water	Total Atoms	Initial Size	Time (μs)
Inner	I-1:2:0:0	I-1:2	80	80	80	-	-	160	160	-	-	142	702	50,440	275,364	163 x 163 x 115	0.5
	I-1:1:0:0	I-1:1	112	112	112	-	-	112	112	-	-	138	698	49,436	264,616	161 x 161 x 115	
	I-2:1:0:0	I-2:1	140	140	140	-	-	70	70	-	-	135	695	48,341	254,563	158 x 158 x 115	
Outer	O-10:5:8:1	O-10:1	80	80	80	64	64	40	40	8	8	493	957	172,779	640,403	150 x 150 x 311	1
	O-10:5:6:2	O-10:2	80	80	80	48	48	40	40	16	16	444	892	156,680	596,328	150 x 150 x 295	
	O-10:4:2:4	O-10:4	80	80	80	16	16	40	40	32	32	445	861	156,237	603,641	148 x 148 x 308	
	O-10:4:1:6	O-10:6	80	80	80	8	8	32	32	48	48	439	855	154,654	619,728	153 x 153 x 301	
	O-10:4:0.5:8	O-10:8	80	80	80	4	4	32	32	64	64	465	913	165,385	682,341	163 x 163 x 296	
	O-10:4:1:10	O-10:10	80	80	80	8	8	32	32	80	80	530	1010	188,979	783,621	172 x 172 x 306	
Asym.	I-1:2-O-10:2		75	75	75	20	20	90	90	10	10	297	762	105,062	433,705	151 x 151 x 210	1
	I-1:2-O-10:6		75	75	75	4	4	86	86	24	24	285	738	101,922	437,925	153 x 153 x 211	
	I-1:2-O-10:10		75	75	75	5	5	86	86	40	40	293	778	106,963	483,432	162 x 162 x 209	
	I-1:1-O-10:2		89	89	89	20	20	69	69	10	10	289	754	103,042	424,248	150 x 150 x 209	
	I-1:1-O-10:6		89	89	89	4	4	65	65	24	24	277	730	99,361	426,845	152 x 152 x 209	
	I-1:1-O-10:10		89	89	89	4	4	65	65	40	40	288	773	104,080	471,392	162 x 162 x 208	
	I-2:1-O-10:2		101	101	101	20	20	51	51	10	10	288	753	102,198	418,816	149 x 149 x 211	
	I-2:1-O-10:6		101	101	101	4	4	47	47	24	24	276	729	98,822	422,328	150 x 150 x 211	
	I-2:1-O-10:10		101	101	101	4	4	47	47	40	40	286	771	104,304	469,162	160 x 160 x 210	

[1]All membrane systems investigated in this study include the symmetric inner leaflet models, the symmetric outer leaflet models with varying LM/LAM ratios, and the asymmetric bilayers combining the fixed inner leaflet compositions with the systematically varied outer leaflet lipoglycan content. The number of each lipid species, counterions, total atoms, and production simulation times are reported for each system.

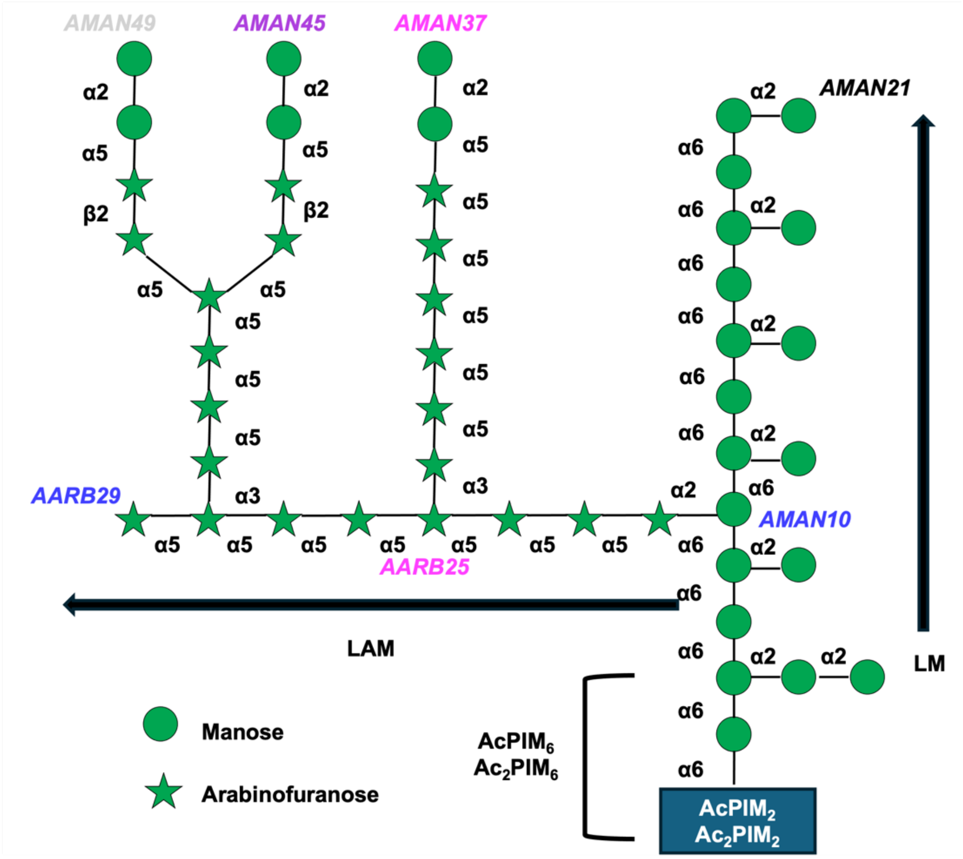

Figure S1. LM/LAM residues used for the tilt angle (AMAN10 and AARB29), averaged distance (AMAN10 and AARB29), and membrane contact (AMAN37, AMAN45, and AMAN49) calculations.

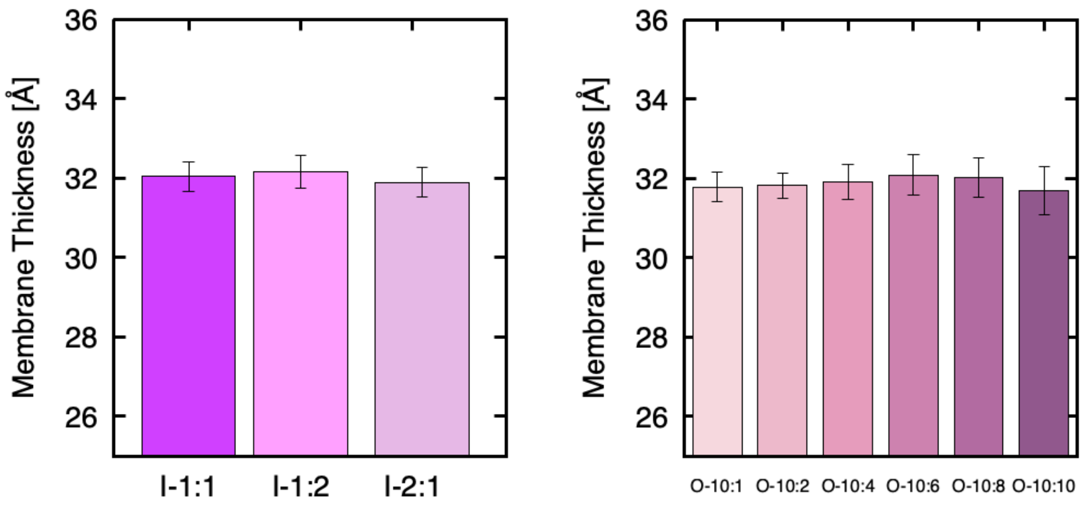

Figure S2. Membrane hydrophobic thicknesses of all symmetric systems.

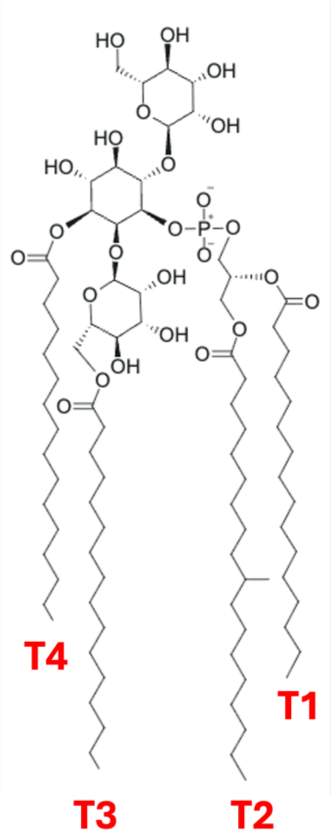

Ac_2PIM_2

Figure S3. Individual chains used for order parameter calculations of LM and LAM.

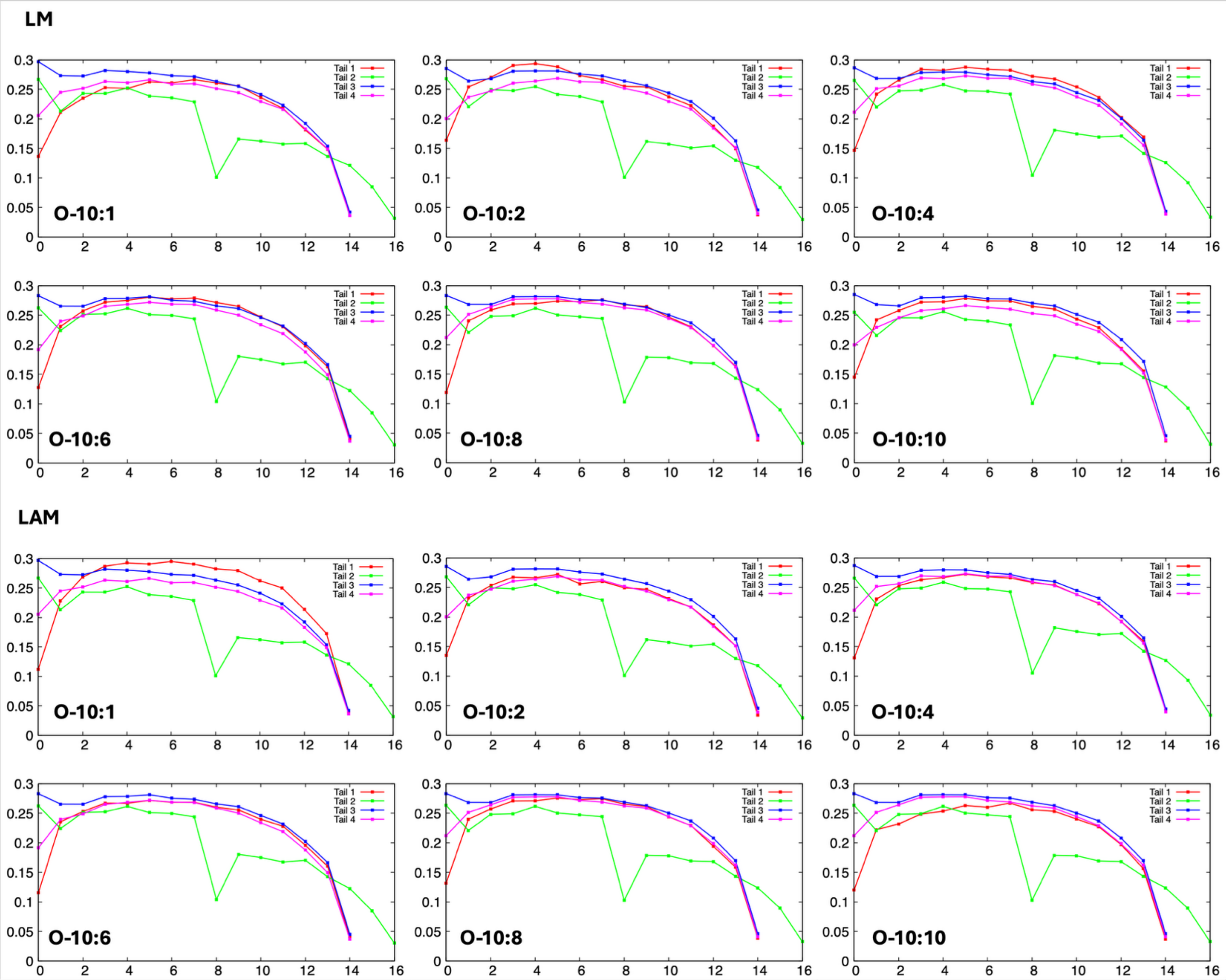

Figure S4. Outer leaflet symmetric order parameters for each carbon in LM and LAM acyl chains in **Figure S3**.
